# In Vitro Bioaccessibility and Bioavailability of Iron from Mature and Microgreen Fenugreek, Rocket and Broccoli

**DOI:** 10.3390/nu12041057

**Published:** 2020-04-10

**Authors:** Kholoud K. Khoja, Amy Buckley, Mohamad F. Aslam, Paul A. Sharp, Gladys O. Latunde-Dada

**Affiliations:** Department of Nutritional Sciences, School of Life Course Sciences, King’s College London, Franklin-Wilkins-Building, 150 Stamford Street, London SE1 9NH, UK; kholoud.khoja@kcl.ac.uk (K.K.K.); amy.buckley@kcl.ac.uk (A.B.); mf.aslam@kcl.ac.uk (M.F.A.); paul.a.sharp@kcl.ac.uk (P.A.S.)

**Keywords:** microgreen, mature, vegetables, minerals, iron

## Abstract

Iron deficiency is a global epidemic affecting a third of the world’s population. Current efforts are focused on investigating sustainable ways to improve the bioavailability of iron in plant-based diets. Incorporating microgreens into the diet of at-risk groups in populations could be a useful tool in the management and prevention of iron deficiency. This study analysed and compared the mineral content and bioavailability of iron from microgreen and mature vegetables. The mineral content of rocket, broccoli and fenugreek microgreens and their mature counterparts was determined using microwave digestion and ICP-OES. Iron solubility and bioavailability from the vegetables were determined by a simulated gastrointestinal in vitro digestion and subsequent measurement of ferritin in Caco-2 cells as a surrogate marker of iron uptake. Iron contents of mature fenugreek and rocket were significantly higher than those of the microgreens. Mature fenugreek and broccoli showed significantly (*p* < 0.001) higher bioaccessibility and low-molecular-weight iron than found in the microgreens. Moreover, iron uptake by Caco-2 cells was significantly higher only from fenugreek microgreens than the mature vegetable. While all vegetables except broccoli enhanced FeSO_4_ uptake, the response to ferric ammonium citrate (FAC) was inhibitory apart from the mature rocket. Ascorbic acid significantly enhanced iron uptake from mature fenugreek and rocket. Microgreen fenugreek may be bred for a higher content of enhancers of iron availability as a strategy to improve iron nutrition in the populace.

## 1. Introduction

Iron deficiency (ID) affects two billion of the world’s seven human billion people, making it the most common nutritional deficiency globally. In particular, ID impacts vulnerable groups such as young children and women of reproductive age [1]. Iron deficiency anaemia (IDA) can irreversibly affect cognitive development during infancy, while poor pregnancy outcomes, decreased work capacity and weakened immunity have all been attributed to IDA during adulthood [2,3,4]. Even in the absence of symptoms, inadequate intakes of vitamins and minerals can have devastating impacts on individuals who, as a result, may not reach their full mental and physical potential [5]. It has, therefore, been proposed that the most sustainable way to prevent IDA is through diets with high iron bioavailability [6]. Consequently, efforts are geared towards the promotion of foods of high iron bioavailability and processes that will enhance the absorption of iron from foods.

There is increasing interest in microgreens, not only as a unique culinary trend but from nutritional, environmental and fiscal viewpoints. Microgreens are vegetables or herbs that are harvested as young seedlings with fully developed cotyledons, but before the first pair of true leaves are completely expanded [7]. With an average height of 2 inches [8], microgreens are to be consumed whole with the stem and cotyledons attached [9]. Microgreens have been reported to contain higher concentrations of antioxidants, vitamins and minerals compared to their mature forms [10].

Broccoli microgreens require approximately 200 times less water and take 95% less time to grow than mature broccoli, and they do not need any application of fertiliser or pesticides [11]. Moreover, broccoli microgreens were reported to have superior levels of Mg, Mn, Cu and Zn compared to the mature vegetable [11]. Furthermore, lettuce microgreens not only contained higher levels of Fe, Ca, Mn, Zn, Se and Mo compared to mature lettuce but also contained lower levels of NO_3_ [12]. The latter is a potentially detrimental form of inorganic N and can lead to methaemoglobinaemia (a decreased ability of the blood to carry oxygen to tissue) when overconsumed [12]. Hence, microgreen vegetables, in addition to their nutritional value, do not pose issues relating to health and safety.

Microgreens may contain higher quantities of promoters of iron absorption such as ascorbic acid. For example, 25 commercially available microgreens of different vegetables were generally found to have higher levels of vitamins and carotenoids than their mature plant counterparts [9]. The authors reported that red cabbage microgreens had twice the ascorbic acid level of mature red cabbage [9]. Moreover, while cooked immature and mature peas had similar levels of iron, bioavailable iron from the immature peas was significantly higher due to the lower levels of phytic acid [13]. However, five commonly consumed brassica microgreen vegetables were shown to have a more extensive range of polyphenols compared to their mature counterparts [14]. While polyphenols are associated with a decreased risk of a variety of chronic diseases [15], they reduce iron absorption in a dose-dependent manner in humans [16]. There is limited information on the mineral profiles and iron bioavailability data from microgreen vegetables. Hence, the current study has analysed the mineral content of rocket, broccoli and fenugreek microgreens and compared these with their mature vegetable counterparts. Furthermore, in vitro iron solubility and iron uptake from microgreen and mature vegetable samples were investigated in Caco-2 cells. These cells were derived from colon adenocarcinoma and are used as a surrogate for enterocytes in the small intestine.

## 2. Materials and Methods 

### 2.1. Reagents and Chemicals

Unless otherwise stated, all the reagents and chemicals used in this study were purchased from Sigma-Aldrich Ltd. (Dorset, UK). Solutions of enzymes were all prepared freshly just before use.

### 2.2. Plant Samples 

Microgreens were cultivated and supplied by Minicrops (Deptford, UK) and were harvested 10 days after planting. Microgreen plants were grown hydroponically using Inserotech. Mineral nutrients were mixed with water at a ratio of 0.5% per litre of water supplied during watering, which occurred once per day. The mineral nutrient solution contained all major and micronutrients. Microgreens were subjected to 120 mmol LED lights for 18-h per day cycles. Mature vegetables were purchased from local markets in London, UK. Microgreen and mature vegetables were washed thoroughly with de-ionised water and dried with paper towels. Subsequently, the vegetables were dried at 37 °C for 72 h. Samples (50 g) of each of the mature or microgreen vegetables were ground in a classic Moulinex AR1043 grinder to fine powders and stored in sealed bags at −70 °C before analysis. 

### 2.3. Moisture Analysis

The moisture content of the samples was determined according to the Association of Official and Analytical Chemists (AOAC) [17] method. Briefly, 2 g of samples were weighed and placed in an oven at 100 °C to dry for 24–48 h until constant weights were achieved. Afterwards, the percentage of moisture content was calculated for each sample.

### 2.4. Determination of Mineral Content in Plant Products

Samples were processed using the MARS 6 Microwave digestion system. Samples (0.5 g of starting material or 5 mL of digest) and 5 mL of concentrated nitric acid were added into reaction vessels and placed into the microwave digester. Digestion of the samples was carried out for an hour. The contents were then transferred into Falcon tubes containing 140 μL of 100 ppm yttrium internal standard, and the volume was made to 14 mL with deionised water. Iron in the samples was read using the inductively coupled plasma optical emission spectrometry (ICP-OES) (Thermo ICAP 6000).

Fractionation of the soluble iron into total bioaccessible Fe (TBF) and low-molecular-weight Fe (LWT) in the digested extracts was carried out by centrifugation and ultrafiltration, as described by Powell et al. [18]. Aqueous suspensions (0.5 mL) were centrifuged (1000 rpm, 5 min), and the supernatant represented the total bioaccessible Fe fraction during in vitro digestion. To separate the low-molecular-weight Fe fraction, a proportion of the supernatant was ultrafiltered through AMICON ULTRA 3 kDa molecular weight cut-off columns (Merck-UFC500396) (1000 rpm, 5 min). Iron concentrations of samples were determined in ICP-OES (Thermo ICAP 6000). The TBF and the LMW were calculated (after subtracting 0.84 ± 0.02 µg/g Fe present in the digestive enzymes) as follows:

[(%) Total bioaccessible Fe] = [(Fe_supernatant after digestion_)/Total Fe] × 100

[(%) Fe low-molecular-weight fraction] = [(Fe_ultrafiltrate_)/Total Fe] × 100

### 2.5. Bioaccessibility Studies: Peptic-Pancreatic in Vitro Digestion

Simulated gastrointestinal digestion was performed on the samples using a procedure described previously [19]. Briefly, in dark tubes, 0.5 g samples were mixed with 10 mL of saline solution (140 mmol/L NaCl and 5 mmol/L KCl) and left for 5 min. Then, the pH was adjusted to 2.0, using 1 M HCl. Afterwards, 0.5 mL of pepsin (Sigma-P7000) (16 mg/mL) was added. Samples were incubated at 37 °C on a rocking platform (150 rpm) for 75 min. Following this, the pH of the samples was adjusted to pH 5.5 using solid NaHCO_3_. Bile extract (Sigma-B8631) and pancreatin (Sigma-P1750) (8.5 mg/mL bile extract and 1.4 mg/mL pancreatin) were added, and the pH adjusted again to pH 7.0. The solution was made up to 30 mL with saline solution, and the samples were incubated at 37 °C for 2 h. At the end of the incubation period, samples were centrifuged at 5000 rpm for 10 min, and the supernatants were decanted and used for the determination of TBF and LMW iron. Thereafter, the digested extracts were applied to Caco-2 cells to estimate iron uptake.

### 2.6. Cell Culture

Human Caco-2 cell line was obtained from American Type Culture Collection (ATCC) at passage 40 and used in experiments at passage 45. Cells were sub-cultured in a 75 cm^2^ flask to 70–80% confluence. The growth medium contained Dulbecco’s Modified Eagle Medium (DMEM), high glucose with glutamine (Sigma, D5796), 10% foetal calf serum (FBS), 1% Penicillin–streptomycin (100×) (Sigma, P4458), 1% L-glutamine (100×) (Sigma, G8540) and 1% MEM non-essential amino acids (Sigma, M7145) in an incubator at 37 °C, 5% CO_2_ and 95% oxygen.

### 2.7. Cell Viability Studies

Caco-2 cells were seeded at a density of 1 × 10^4^ cells/cm^2^ in 96-well plates. After 14 days of differentiation, the medium was discarded, and the cells were washed twice with sterile phosphate-buffered saline (PBS) and then incubated with 100 µL of the digested extracts of fenugreek, rocket and broccoli mature or microgreen for 2 h. Following this, 100 µL of fresh Modified Eagle’s medium (DME) (Sigma, D1145) along with 10 µL of Dimethylthiazol-2-yl)-2,5-diphenyltetrazolium bromide (MTT) (Sigma, M2003) sterile solution (5 mg/mL MTT in PBS) were added to each well. After incubating for 3 h in the dark at 37 °C, 100 μL of a solubilisation buffer in dimethyl sulfoxide(DMSO) was added and incubated for 15 min at room temperature. To determine the MTT reaction in the cells, optical density was read in a microplate reader (Bio-Tek ELx800) at 490 nm. Cell viability was expressed as a percentage of the controls.

### 2.8. Iron Bioavailability Studies

Human Caco-2 cell line was used to evaluate the bioavailability of iron. These cells are derived from colon adenocarcinoma and are used as a surrogate for enterocytes in the small intestine. This model compares well with human studies and is commonly used to analyse iron bioavailability from various food types [19]. Caco-2 cells were trypsinised and cultured in 6-well plates for 14 days to allow them to differentiate, and the medium was changed every 2 days. Before experiments, cells in 6-well plates were treated with 2 mL serum-free medium (SFM) MEM (Sigma-M4655) for 24 h. Sample digests were centrifuged and heated at 100 °C for 5 min to inactivate the digestive enzymes. Serum-free medium (1 mL) was added to cells, followed by the addition of 1 mL of sample digest, and these were incubated in a rotating shaker for 2 h. Following this, 1 mL of MEM was added, and samples were incubated at 37 °C for a further 22 h for ferritin synthesis. After the incubation, the culture medium was discarded, and cells were washed with versene (in PBS + EDTA). Afterwards, 100 µL of mammalian protein extraction reagent (MPER, Thermo Scientific, Waltham, MA, USA) was added to wells and left on a shaker for 15 min for cell lysis.

Ferritin ELISA kit, Spectro Ferritin MT (Ramco Laboratories Inc., Stafford, TX, USA) was used to determine ferritin content in the cells according to the manufacturer’s protocol.

### 2.9. Phytic Acid Analysis

Phytic acid content (total phosphorus) was measured by using the Megazyme (Megazyme- K-PHYT, Bray, Ireland) kit and following the protocol described by the manufacturer [20]. Briefly, acid extracts of inositol phosphates from the samples were digested with phytase and alkaline phosphatase suspension was used to release phosphate from all the myo-inositol phosphate forms. The total phosphate released was measured using a modified colourimetric method, and it was calculated as grams of phosphorus per 100 g of sample material.

### 2.10. Statistical Analysis

Experiments were performed in 3–6 replicates and data are shown as mean ± standard error of the means (SEM). Comparisons of iron content, solubility and ferritin concentrations in Caco-2 cells were analysed using one- or two-way ANOVA followed by Tukey’s post hoc test where appropriate, using GraphPad Prism software. The significance level was at *p* ≤ 0.05.

## 3. Results

### 3.1. Mineral Content and Moisture in Plant Products

Moisture content did not vary between the mature and microgreen plant food samples (range 86.97–92.6%). There were significant differences (*p* ≤ 0.05) in some of the mineral content between the mature and microgreens of some of the vegetables (Table 1). Mature fenugreek had significantly higher amounts of Fe, Ca, Mg Mn and Mo than the fenugreek microgreen. Moreover, mature rocket contained significantly higher levels of Fe and Ca, Cu, Mg and Mn than the microgreen. In addition, mature broccoli contained significantly higher levels of Cu and Zn than the microgreen (Table 1).

### 3.2. The Bioaccessible and Fractional Low-Molecular-Weight Iron Content of the Digested Extracts of the Vegetables

In vitro simulated peptic-pancreatic digestion was then conducted to estimate the percentage of total bioaccessible iron (TBF) and low-molecular-weight (LMW) iron fraction from the vegetable samples. The absolute amount µg/g and percentage of Fe and the TBF in the mature fenugreek and broccoli were significantly (*p* ≤ 0001) higher than in the microgreen. The LMW amount µg/g of Fe in the rocket microgreen was significantly higher than in the mature vegetable. LMW Fe was significantly higher in mature broccoli than in the microgreen vegetable (Table 2).

### 3.3. Cell Viability of Caco-2 cells after Exposure to Digested Vegetable Samples

**Figure 1:**
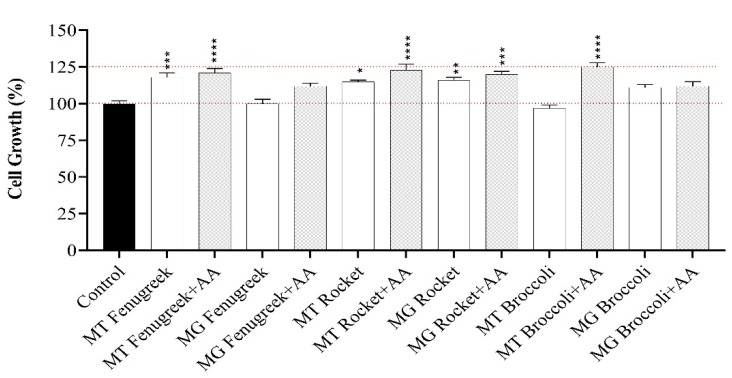
Cell viability of Caco-2 after exposure to digest vegetable Mature (MT) and Microgreen (MG) samples alone or with added ascorbic acid (AA) during digestion for 2 h. Results are presented as means ± SEM, *n* = 4. Data were analysed using a one-way ANOVA test. Different letters on top of bars indicate a significant difference: * (*p* ≤ 0.05), ** (*p* ≤ 0.01), *** (*p* ≤ 0.001) and **** (*p* ≤ 0.0001).

To ascertain whether the digested extracts from the plants were not cytotoxic to Caco-2 cells, MTT viability assay was performed. The application of the boiled samples of the digested extracts of fenugreek, rocket and broccoli mature and microgreen, with and without ascorbic acid (AA), did not adversely affect the viability of Caco-2 cells. Indeed, in some cases, there was a significantly positive effect on cell growth (Figure 1).

### 3.4. In Vitro Bioavailability of Iron from Fenugreek, Rocket or Broccoli in Caco-2 Cells

**Figure 2:**
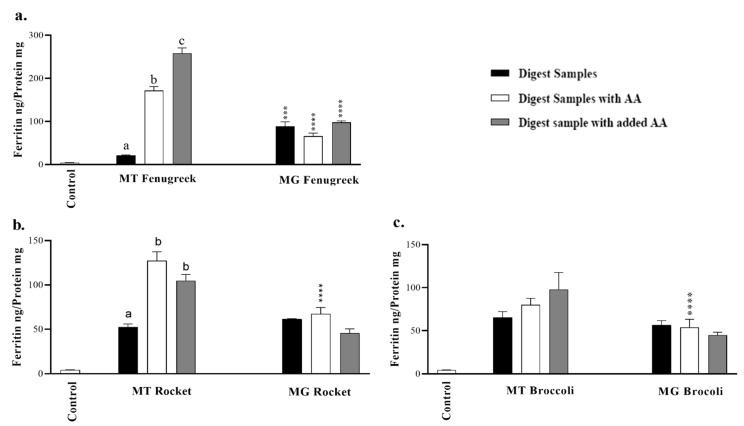
Iron uptake by Caco-2 cells from (**a**) Fenugreek, (**b**) Rocket, and (**c**) Broccoli mature (MT) and microgreen (MG) vegetables alone, with added ascorbic acid (AA) during digestion and vegetable samples with AA added during exposure to cells. The control treatment represents ferritin formation in Caco-2 cells in the presence of extract containing only the digestive enzymes. Results are presented as means ± SEM, *n* = 6. Data were analysed using a two-way ANOVA test. Different letters on top of bars indicate significant differences (*p* ≤ 0.05) between data in the same group. The differences between MT and MG groups of the same vegetables are denoted: * (*p* ≤ 0.05), ** (*p* ≤ 0.01), *** (*p* ≤ 0.001) and **** (*p* ≤ 0.0001).

To estimate the bioavailability of iron from the samples, an in vitro simulated peptic–pancreatic digestion was carried out followed by ferritin analysis (a surrogate marker for iron absorption) in Caco-2 cells. The cell baseline ferritin formation in Caco-2 cells that were treated with MEM medium alone was 4.2 ± 0.4 ng/mg protein. Fenugreek microgreen exhibited comparatively higher iron bioavailability than the mature vegetable (Figure 2a). There were no differences in the bioavailability of Fe from mature and microgreen rocket and broccoli.

To enhance iron extraction during the peptic-pancreatic digestion of the mature and microgreen fenugreek, rocket and broccoli, ascorbic acid was added to the samples during the digestion process. With the exception of mature fenugreek and rocket, extracts displayed no enhancing effect of adding ascorbic acid during in vitro digestion on iron uptake in Caco-2 cells (Figure 2). Moreover, ascorbic acid significantly (*p* ≤ 0.001) enhanced iron uptake from mature fenugreek and rocket when added directly to Caco-2 cells. 

### 3.5. Modulating Effects of Fenugreek, Rocket and Broccoli on Iron Bioavailability from Iron Salts in Caco-2 Cells

**Figure 3:**
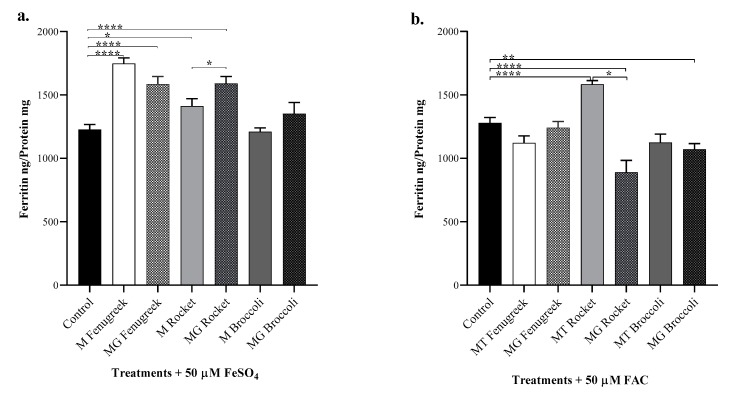
Iron uptake by Caco-2 cells from vegetable digests. Cells were exposed to the digest samples with added 50 µM: (**a**) FeSO_4_ (Fe(II)); and (**b**) FAC (Fe(III)). Results are presented as means of ± SEM *n* = 6. Data were analysed using a two-way ANOVA test between control and vegetable samples or among mature and microgreen samples. The differences between groups are denoted: * (*p* ≤ 0.05), ** (*p* ≤ 0.01) and **** (*p* ≤ 0.0001).

We next explored the interactions and the modulating effects of the components of the plant products on the bioavailability of iron salts. FeSO_4_ (Fe(II)) and FAC (Fe(III)) had comparable iron availability for uptake in Caco-2 cells (Figure 3). Except for broccoli, the other plant samples significantly (*p* ≤ 0.05) enhanced iron bioavailability from FeSO_4_. With FAC, however, iron bioavailability was enhanced by mature rocket only while microgreen rocket and broccoli were significantly (*p* ≤ 0.05) inhibitory to FAC uptake.

### 3.6. Phytic Acid Levels in Fenugreek, Rocket and Broccoli

**Figure 4:**
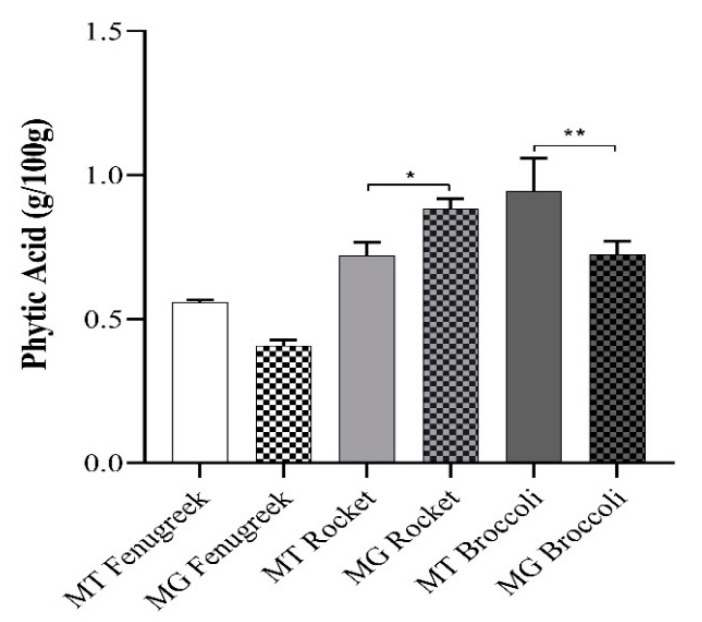
Phytic acid content (g/100 g) of the vegetable samples. Values are presented as means ± SEM, *n* = 6. Comparison of means of microgreens and mature vegetables were analysed using an independent samples t-test. The differences between the groups are denoted: * (*p ≤* 0.05) and ** (*p ≤* 0.01).

Phytic acid is a major inhibitory component in plant foods. Figure 4 shows a significant level of variations in the phytate content of the plant products. Differences in the phytic acid levels did not correlate with the bioaccessibility and bioavailability of fenugreek and broccoli vegetables. Microgreen rocket, with a higher level of phytic acid, displayed less iron uptake from FAC than the mature form (Figure 3b).

## 4. Discussion

Diversification of plant-based diets is being proposed as a sustainable way to increase intake of bioavailable iron in populations at risk of iron deficiency. According to Franks [21], microgreens are simple and cheap to grow in a household setting and could be sources of vital micronutrients in the diets of at-risk groups within a populace. While the nutrient profiles of some microgreens have been reported, to our knowledge, this is the first study to assess the bioavailability of iron in microgreen vegetables compared to their mature form.

The present study found that broccoli microgreens, cultivated hydroponically, had significantly lower levels of some minerals than mature vegetables (Table 1). These findings are in contrast to a previous report that showed that hydroponically grown broccoli microgreens had higher levels of Zn than mature broccoli [11]. Moreover, compost-grown microgreens had significantly higher levels of Fe, P, K, Ca and Na [11], while Fe, Ca, Cu, Mg, Mn and Zn levels in broccoli microgreens were higher than the findings in the current study [7]. It is interesting to note that the microgreens investigated by Xiao were grown on peat moss and this could account for the differences observed in the findings as it was reported that cultivation practices significantly impacted the mineral composition of broccoli microgreens [11]. As microgreens could also be cultivated in soils, and even with hydroponics, factors such as the composition of the fertilisers, vegetable mats, vermiculite or pebbles support substrates could introduce high variability. Mature vegetable mineral content in the current study was similar to those reported in McCance and Widdowson’s, ‘The Composition of Foods’ [22].

Iron in the digest during the in vitro simulated digestion is described as total bioaccessible (TBF), and LWT iron fractions and the proportion that is absorbed by cells or the intestine is bioavailable [23]. There was a significant difference in the percentage of TBF between mature vegetable and microgreen counterparts (Table 2). Respective TBF and LWT from fenugreek and broccoli were higher from mature than the microgreens.

Of all the vegetables employed in the current study, only fenugreek microgreen had a significant increase in iron uptake compared to mature fenugreek sample (Figure 2a). Previous work showed that iron uptake by Caco-2 cells was significantly higher from immature peas than mature, which was attributed to lower phytic acid content of immature peas [13]. Similarly, iron bioaccessibility reported for immature and mature peas were not significantly different [13]. Promoters and inhibitors in the foods, pH and particle size [24] are all factors that influence iron solubility. There was no significant difference in iron uptake of the microgreen and mature forms of both rocket and broccoli (Figure 2b,c). All microgreen samples had significantly less iron than their mature counterparts. It is known that the bioavailability of Fe from foods can be highly variable and it is dependent on the interactions of various dietary inhibitors and promoters of absorption as well as the iron status of the individual [25]. Although mature fenugreek was reported to have the highest tannin level and lower ascorbic acid content compared to several other green leafy vegetables, it exhibited a high percent ionisable iron [25], nonetheless.

Furthermore, the calcium to iron ratios, respectively, for fenugreek, rocket and broccoli are 247.21, 280.94 and 71.94 (mature) and 93.04, 302.04 and 457.95 for the microgreens. Broccoli microgreens contained significantly more calcium than mature broccoli (Table 1) which may explain the reduced iron uptake by Caco-2 cells (Figure 2c). Calcium exerts a dose-dependent inhibitory effect on iron absorption [26]. Interestingly, only fenugreek showed significant iron uptake by Caco-2 cells without exogenous ascorbic acid (Figure 2a). However, human trials reported that processing of plant foods to increase iron availability by reducing endogenous inhibitors of iron absorption was not sufficient to improve the iron status of infants compared to unprocessed placebo [27].

The ratio of phytate to iron has been shown to correlate with iron dialysability [28]. The phytate to iron ratios respectively for fenugreek, rocket and broccoli are 51.43, 55.18 and 173.62 (mature) and 49.57, 118.64 and 129.72 for the microgreens. It appears that processing in the study did not sufficiently reduce phytate levels to improve iron uptake in vivo significantly [28]. In the presence of ascorbic acid, iron uptake by Caco-2 cells from mature fenugreek and rocket were significantly higher compared to the microgreen (Figure 2a,b). As expected, ascorbic acid significantly enhanced iron uptake from FeSO_4_ by Caco-2 cells (Figure S1).

Ascorbic acid is a potent promoter of iron uptake, acting by reducing ferric Fe to ferrous Fe [29], maintaining iron in solution and has been reported to reverse the inhibition of iron uptake by polyphenols [30]. On the other hand, vitamin C in plant products are 87.49 and 79, 20 and 154.82 and 89.3 and 32.7 mg/100 g for mature and microgreen fenugreek, rocket and broccoli, respectively [31]. However, the beneficial effect of ascorbic acid can be neutralised by high phytochemical levels in fruit and vegetables [32]. For example, 50 mg ascorbic acid was required to attenuate the inhibitory influence of 100 mg tannic acid on iron absorption [33]. An inverse relationship was demonstrated between the polyphenol content of vegetables and iron absorption [30]. Hence, microgreen vegetables are abundant in both potent inhibitors and an enhancer of iron availability. In general, the comparatively low solubility of iron from plant products may be due to chemical complexation with a range of inhibitory factors including phytate, polyphenols and fibre [32].

It has been suggested that green leafy vegetables could be used as a natural iron fortificant to improve iron bioavailability of typical Indian meals [34]. Although cooked fenugreek was not highly rated as a fortificant, the addition of a raw fenugreek salad with lemon juice to a meal significantly increased bioavailable iron content [34]. While blanching of green leaves reduces phytic acid content [35], this can induce ascorbic acid losses [36]. Thus, the authors concluded that the addition of a raw green leafy salad dressed with a natural source of ascorbic acid, such as lemon juice, is a cost-effective way for individuals to increase the bioavailable iron content of their meals and sufficiently meet their daily requirements [34]. Moreover, other factors found in green leafy vegetables may act as enhancers of iron bioavailability. For example, fructose 1,6-biphosphate, present at high levels in cabbage, has been shown to increase iron uptake in Caco-2 cells [37]. Potentially, incorporating microgreen vegetables in their dried forms into various recipes could make approximately 50% contribution per 100 g portion to the recommended daily iron intake in the diet [38]. For example, the reference nutrient intake (RNI) for iron in the UK is 14.8 mg per day for adult women. Adding 25–50 g dried microgreen vegetables (average content 7 mg/100 g of the three microgreen vegetables) to a 100 g portion of vegetable casserole or salad, sprinkled with lemon (as a taste enhancer and a rich source of vitamin C) would contribute approximately 24–47% of RNI or 0.37–0.74 mg iron.

It will be crucial to determine the content of endogenous inhibitors (phytic acid, tannic acid and total polyphenol content) and promoters (ascorbic acid and fructose 1,6-biphosphate) of iron absorption in these vegetable samples to understand their potential as sources of bioavailable iron. Fenugreek microgreens revealed significantly more iron uptake in Caco-2 cells than mature fenugreek and could be promoted as a salad to improve iron status in groups that are vulnerable to iron deficiency. Potentially, mineral levels of hydroponic solutions used to grow microgreen vegetables could be manipulated to increase the mineral content of the vegetables. Plant breeding practices could also be geared towards the selection of microgreens with higher endogenous levels of ascorbic acid. Further research is also necessary to investigate methods of preparation, palatability and sensory characteristics of microgreen vegetables in both their fresh form and also dried powders which could be used to enhance iron bioavailability of vegetable dishes. Moreover, human intervention trials to investigate iron absorption from microgreens are highly recommended. 

## Figures and Tables

**Table 1:** Moisture and mineral content in the dried vegetable samples (µg/g).

Samples	Fenugreek		Rocket		Broccoli	
	Mature	Microgreen	Mature	Microgreen	Mature	Microgreen
Moisture (%)	86.97	90.52	92.6	92.28	89.89	92.12
Fe	108.5 ± 0.76	82.32 ± 1.22 ****	130.82 ± 82.3	74.41 ± 1.21 ****	54.37 ± 0.48	55.90 ± 1.03
Ca	26834.2 ± 139.56	7658.93 ± 79.58 ****	36753.4 ± 319.08	22475.13 ±137.14 ****	3911.91 ± 35.9	25601.7 ± 136.28 ****
Cu	6.72 ± 0.22	9.15 ± 0.11 ****	11.65 ± 0.32	9.93 ± 0.40 **	8.26 ± 0.42	6.40 ± 0.12 ***
Mg	2687.4 ± 18.31	1645.0 ± 24.92 ****	3886.85 ± 45.66	3472.46 ± 140.1 ***	1752.92 ± 9.52	3702.53 ± 52.35 ****
Mn	41.24 ± 1.4	24.94 ± 0.37 ****	119.93 ± 1.22	92.30 ± 1.10 ****	25.67 ± 0.20	115.0 ± 1.15 *
Mo	6.18 ± 0.025	2.88 ± 0.05 ****	3.23 ± 0.036	7.91 ± 0.29 ****	1.67 ± 0.007	2.76 ± 0.046 ****
Zn	23.27 ± 0.32	34.92 ± 0.52 ****	80.29 ± 0.03	90.34 ± 1.58 ****	52.22 ± 0.22	39.65 ± 0.67 ****

Mineral contents, calcium (Ca), copper (Cu), iron (Fe), magnesium (Mg), manganese (Mn), zinc (Zn) and Molybdenum (Mo) in 100 g of sample. Results are presented as means ± SEM, *n* = 6. Comparison of means of microgreens and mature vegetables were analysed using an independent samples t-test. Different * indicates a significant difference: * (*p* ≤ 0.05), ** (*p* ≤ 0.01), *** (*p* ≤ 0.001) and **** (*p* ≤ 0.0001).

**Table 2:** Total bioaccessible and low-molecular-weight iron in digest samples.

Samples		Fenugreek		Rocket		Broccoli	
		Mature	Microgreens	Mature	Microgreens	Mature	Microgreens
Total Bioaccessible Iron (TBF)	µg/g	61.07 ± 0.11	34.27 ± 0.11 ****	25.02 ± 0.07	28.15 ± 0.64 ****	24.73 ± 0.05	20.10 ± 0.02 ****
	%	56.26 ± 0.10	41.63 ± 0.13 ****	19.13 ± 0.06	37.83 ± 0.87 ****	45.49 ± 0.10	35.96 ± 0.31 ****
Low-Molecular-Weight Iron (LMW)	µg/g	6.32 ± 0.11	5.76 ± 0.11	7.29 ± 0.16	5.44 ± 0.08 ****	6.00 ± 0.11	4.41 ± 0.06 ****
	%	5.82 ± 0.10	7.00 ± 0.14	5.58 ± 0.13	7.32 ± 0.12	11.04 ± 0.20	7.88 ± 0.11 **

Results are presented as means of ± SEM, *n* = 3. Comparison of means of microgreens and mature vegetables were analysed using an independent samples t-test. ** (*p* ≤ 0.01), and **** (*p* ≤ 0.0001).

## Supplementary Materials

The following are available online at https://www.mdpi.com/2072-6643/12/4/1057/s1, Figure S1: Iron uptake by Caco-2 cells from ferrous sulfate. Samples FeSO4 (50 μM) iron salts alone with or ascorbic acid (AA) were exposed to cells directly or after in vitro digestion process.
