# Supplementary material for: In Vitro Bioaccessibility and Bioavailability of Iron from Mature and Microgreen Fenugreek, Rocket and Broccoli
Source: Nutrients. 2020 Apr 10;12(4):1057. doi: 10.3390/nu12041057 (PMC7231393; doi:10.3390/nu12041057)
Supplement: Supplementary file 1 [file nutrients-12-01057-s001.pdf]

nutrients

MDPI

Supplementary Data -1

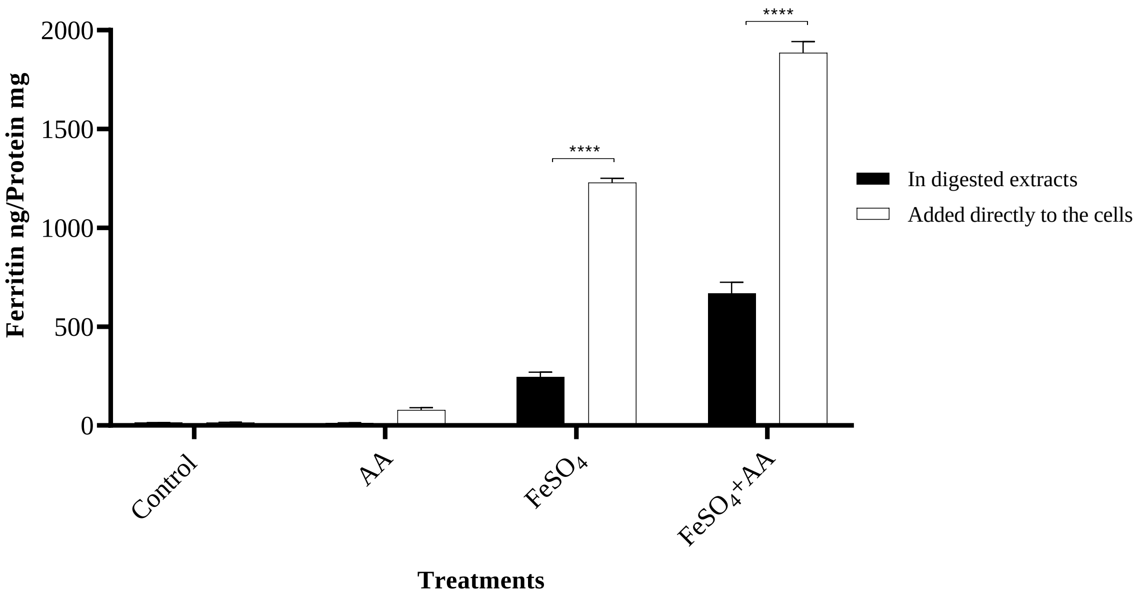

Figure 1: Iron uptake by Caco-2 cells from ferrous sulfate. Samples FeSO4 (50 μM) iron salts alone with or ascorbic acid (AA) were exposed to cells directly or after in vitro digestion process. The control treatment represents ferritin formation in Caco-2 cells in the presence of extract containing only the digestive enzymes. Results are presented as means of n ± SEM, n= 3. Data were analysed using a two-way ANOVA. Significant differences (P≤0.05) are between samples that were subjected to the digestion protocol and those added directly to cells are denoted ****(P≤0.0001).
